# Neural Correlates of Oral Stereognosis—An fMRI Study

**DOI:** 10.1007/s00455-022-10517-2

**Published:** 2022-09-10

**Authors:** Beate Schumann-Werner, Sinika Schaefer, Silja Schramm, Harshal Jayeshkumar Patel, Ferdinand Christoph Binkofski, Cornelius Johannes Werner

**Affiliations:** 1 Department of Neurology, Medical Faculty, RWTH Aachen University, Pauwelsstrasse 30, 52074 Aachen, Germany; 2 Department of Neurology and Geriatrics, Johanniter Hospital Stendal, Wendstrasse 31, 39576 Hansestadt Stendal, Germany; 3 Section Clinical Cognitive Sciences, Department of Neurology, Medical Faculty, RWTH Aachen University, Pauwelsstrasse 30, 52074 Aachen, Germany

**Keywords:** Oral stereognosis, Dysphagia, fMRI, Neural correlates, Manual stereognosis

## Abstract

Oral stereognosis is the ability to recognize, discriminate and localize a bolus in the oral cavity. Clinical observation indicates deficits in oral stereognosis in patients with vascular or neurodegenerative diseases particularly affecting the parietal lobes. However, the precise neural representation of oral stereognosis remains unclear whereas the neural network of manual stereognosis has already been identified. We hypothesize that oral and manual stereognosis share common neuronal substrates whilst also showing somatotopic distribution. Functional magnetic resonance images (fMRI; Siemens Prisma 3 T) from 20 healthy right-handed participants (11 female; mean age 25.7 years) using a cross-modal task of oral and manual spatial object manipulation were acquired. Data were analyzed using FSL software using a block design and standard analytical and statistical procedures. A conjunction analysis targeted the common neuronal substrate for stereognosis. Activations associated with manual and oral stereognosis were found in partially overlapping fronto-parietal networks in a somatotopic fashion, where oral stereognosis is located caudally from manual stereognosis. A significant overlap was seen in the left anterior intraparietal sulcus. Additionally, cerebellar activations were shown particularly for the oral condition. Spatial arrangement of shaped boli in the oral cavity is associated with neuronal activity in fronto-parietal networks and the cerebellum. These findings have significant implications for clinical diagnostics and management of patients with lesions or atrophy in parietal lobule (e.g. Alzheimer’s disease, stroke). More studies are required to investigate the clinical effect of damage to these areas, such as loss of oral stereognosis or an impaired oral phase.

**Supplementary Information:**

The online version contains supplementary material available at 10.1007/s00455-022-10517-2.

## Introduction

Stereognosis is the ability to perceive and recognize the form of an object in the absence of visual and auditory information. Crucially, the loss of stereognosis (astereognosis) can occur in preserved tactile sensation of the hand. This was first observed by Carl Wernicke [1], who called it “kortikale Tastlähmung” (cortical paralysis of touch), and was later replicated by others [2–4]. Oral stereognosis has been recognized much later [5]. Impaired oral stereognosis has been associated with impaired oral swallowing function in children, previously [6, 7]. It seems plausible, therefore, that oral stereognosis could be an essential component of proper bolus handling in general. Oral stereognosis can be impaired in stroke [8] and thus might hypothetically contribute to stroke-associated dysphagia. Here, it has received very little attention, however, with just two studies examining this phenomenon [9, 10]. In these studies, the authors found that while stroke patients exhibit this phenomenon, patients suffering from Parkinson’s disease do not. Later studies confirmed that in Parkinson’s disease there are no differences in oral stereognosis to healthy controls [11], and that there is no influence of clinical ON or OFF state on oral stereognostic abilities [12]. This, of course, prompts the question on the cerebral representation of oral stereognosis.

The exact neuronal correlates of this ability have not been studied extensively. The majority of previous imaging studies focused on oral motor functions, for instance jaw and tongue movements or chewing, or on oral sensation, as reviewed by [13]. Only one prior study employing functional magnetic resonance imaging (fMRI) has investigated the neural correlates of oral stereognosis so far [14]. In this study, oral and manual stereognosis both elicit activation in mainly identical cerebral regions. The only other neurophysiological study dealing with neural correlates of oral stereognosis employed near infrared spectroscopy (NIRS, [15]), where anatomical precision is not provided by the method. Therefore, the neural correlates of isolated oral stereognosis remain unclear.

In contrast to oral stereognosis, the neural network of manual stereognosis has already been described precisely (e.g. [16], review in [17]). In the study by Binkofski et al., a fronto-parietal network including anterior intraparietal sulcus, SII and ventral premotor cortex (vPMC) has been identified as the neural basis of manual stereognosis. In particular, anterior intraparietal sulcus seems to subserve supramodal aspects of shape recognition [18]. Furthermore, it has already been shown that lesions in parietal areas typically cause impaired manual stereognosis [19].

With respect to oral stereognosis, which is pertinent to healthy swallowing, the precise anatomical correlates have not been sufficiently elucidated. Thus, we designed and conducted a fMRI experiment specifically targeting the neural correlates of oral stereognosis employing strictly controlled conditions. Based on previous studies, we hypothesize that oral and manual stereognosis share common neuronal substrates for supramodal processing located mainly in anterior parietal and prefrontal cortex, whilst perhaps also showing a somatotopic distribution.

## Methods

### Participants

The imaging study included 20 healthy participants (11 females, 9 males; mean age 25.7 years; range 20.6–34.6 years), who were recruited at Rheinisch-Westfälische Technische Hochschule (RWTH) Aachen University. All participants had normal or corrected-to-normal vision, normal dentition and no history of neurological disorders. They were screened by a neurologist for medical issues that could affect oral sensation including smoking. All participants were right-handed, as assessed by the Edinburgh Handedness Inventory [20]. They gave written informed consent to the study protocol. All procedures followed safety guidelines for MRI (magnetic resonance imaging) research at the Laboratory of Interdisciplinary Center for Clinical Research (Interdisziplinäres Zentrum für Klinische Forschung, IZKF). The study was approved by the Ethics Committee of the Medical Faculty, University Hospital RWTH Aachen (EK083/16).

### Test Pieces

**Fig. 1:**
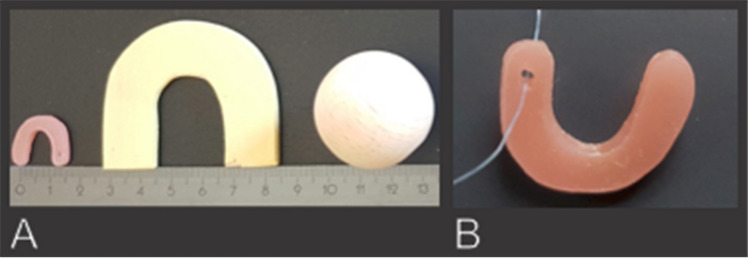
Test pieces. Test piece for the oral experimental task (**a**). Test piece for the manual experimental task (**b**). Test piece for the manual control task (**c**). Visual stimulus (**d**)

For the oral experimental task, we used a u-shaped, tasteless, and smooth test piece (17 × 12 × 2 mm; Koch Zahntechnik GmbH; dental technology; Düsseldorf, Germany), made of autopolymerizing acrylic resin (Fig. 1). The test piece is part of a set of 9 test items which has been originally developed to screen for oral stereognosis in children with orofacial myofunctional disorders [21]. We used the u-shaped test piece because it could be spatially arranged in four different directions. A nylon thread was attached to the test piece to avoid involuntary swallowing. For the manual experimental task, we used a u-shaped, wooden, and smooth test piece (5 × 4.5 × 0.3 cm). For the manual control task, we used a smooth wooden sphere with a diameter of 3.5 cm (Fig. 1). Here, we followed the example of Binkofski et al. [22] who also used slightly differently sized objects for stereognosis and control conditions. Both test pieces for the manual task were chosen larger than for the oral task because a test piece of equal size would have been difficult to arrange in the participant’s hand or mouth, respectively.

### Procedure and Task

#### Pre-Testing (*n* = 8)

Prior to imaging, we performed a behavioural experiment with *n* = 8 participants in which we defined the duration of stimulus-presentation for the fMRI experiment. Here, the test piece was placed onto participants’ dorsum of the tongue in horizontal position with opening to the front. Participants were in lying position, while twenty visual stimuli in different spatial positions were introduced on a 30 inch laptop (40 cm distance). Participants were instructed to arrange the test piece into the position shown on the laptop screen (visual stimulus). They indicated with their thumb when they had arranged the test piece for oral stereognosis in the presented position and stuck out their tongue to verify the position. The duration needed was measured by stopwatch. Then, the next stimulus was presented.

#### fMRI Experiment (*n* = 20)

Prior to scanning, participants were familiarized with the symbols presented during the experiment and the test pieces. Moreover, each experimental and each control task was performed once outside the scanner in order to ensure correct performance.

**Fig. 2:**
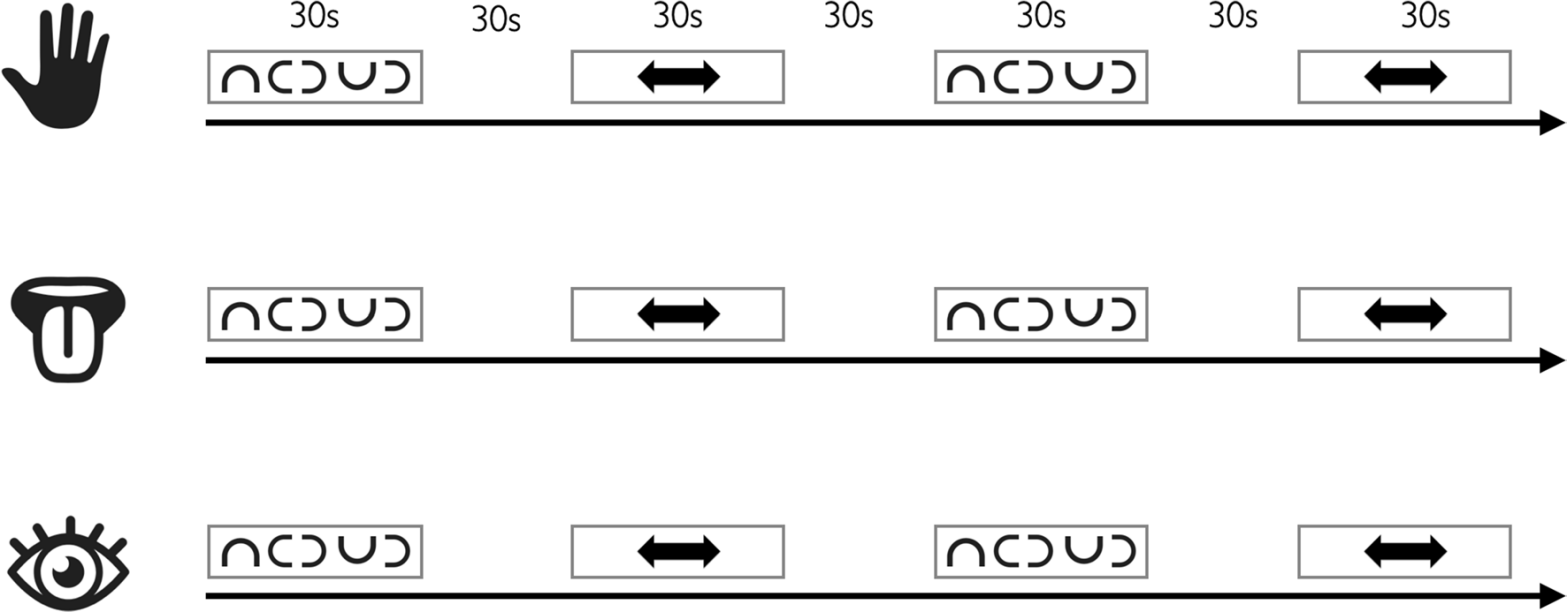
Study design of the three functional runs (oral stereognosis, manual stereognosis, visual control run). Block with u-shapes = experimental block; bidirectional arrow = control block; Each run consisted of four experimental and four control blocks which were presented alternately

The fMRI experiment consisted of three functional runs (oral stereognosis, manual stereognosis, and one visual control run). Each run consisted of four experimental blocks and four control blocks. Each block lasted 30 s and was followed by a 30 s baseline. In total, the duration of each run was 510 s (Fig. 2).

For all conditions, head fixation and noise protection were used to reduce head motion and protect participants’ hearing. During the 30 s baselines the participants were told to look at the fixation cross, to relax their mind, and to avoid body moving. During the experimental blocks, five visual stimuli were presented in pseudo-randomized order (see file 1 of the Supplementary Material). According to the behavioural results, visual stimuli were presented for six seconds each, for a total of 30 s of stimulation. The visual stimuli and symbols were presented on a screen behind the 3 T MRI projected to a mirror above the participants’ head inside the MRI.

##### Run 1: Oral Stereognosis

Prior to the oral stereognosis run, the test piece was placed onto the participants’ dorsum of the tongue and the affixed nylon thread was taped to the participants’ collarbone. Participants were instructed to arrange the u-shaped test piece in their mouths using the tongues according to the direction indicated by the instructional stimulus.

In the control blocks, which also lasted 30 s each, a control symbol () was shown, instructing the subjects to move the test piece inside their mouthes from the left to the right using lateral tongue movements in the cavum oris, giving no attention to the orientation of the test piece.

##### Run 2: Manual Stereognosis

For the manual stereognosis run, the u-shaped test piece and the wooden sphere were placed into the participants’ right hands in an alternating fashion. Test pieces were removed and replaced by one of the experimenters inside the scanner room. For the stereognosis run, the same visual stimuli were used as in the oral stereognosis run. Here, participants were requested to arrange the wooden u-shape in the same direction as indicated on the screen and show it to the experimenter. Similarly, in the control run, subjects were presented with the according symbol and were requested to simply manipulate (e.g. rotate) a wooden sphere in their right hands.

##### Run 3: Visual Stimulation

As a visual control run, we presented the identical visual presentation stimuli which were already shown in the previous functional runs (spatially arranged test items vs. control condition arrows in an alternating manner). During scanning participants were instructed to merely watch the presentation. From this setup, six conditions resulted: oral stereo (OS), oral control (OC), manual stereo (MS), manual control (MC), visual stereo (VS) and visual control (VC).

### Data Acquisition and Image Analysis

#### MRI Data Acquisition

MRI and fMRI measurements were performed on a Siemens Prisma 3 T MRI utilizing standard sequences. T1-weighted images (corresponding to 176 sagittal slices, MPRAGE, TR = 1.900 ms, TE = 2.5 ms, matrix size = 256 × 246, voxel size = 1 × 1 × 1 mm^3^, FoV = 250 × 250 mm^2^) served as an anatomical reference for the functional images. Furthermore, whole-brain coverage gradient echo EPI imaging was conducted (TR = 2.200 ms, TE = 30 ms, slice thickness = 3.1 mm, matrix size = 64 × 64, FoV = 200 mm × 220 volumes) for functional imaging purposes. The first three volumes were discarded prior to analysis to allow for stabilization of the magnetic field.

#### MRI Data Analysis

On the single subject level, pre-processing was performed using the statistical parametric mapping software FEAT (v.6.00, FMRIB’s Software Library, www.fmrib.ox.ac.uk/fsl). Functional images of all subjects were motion corrected and temporally filtered (sigma = 30 s), realigned and co-registered to the participants’ high resolution T1-image. Finally, the images were normalized by matching the standardized MNI152-template (Montreal National Institute) using FLIRT (FMRIB’s linear image registration tool) and FNIRT (FMRIB’s nonlinear image registration tool). Spatial smoothing [FWHM = 5 mm]) was performed. On the first level, we calculated the following contrasts: OS > OC, MS > MC and VS > VC.

Utilizing parameter estimates from contrast VS > VC, we created a mask comprising all significant voxels on the group level using *z* > 2.3 and a cluster-corrected *p*-value of < 0.05.

Second level analyses for contrasts OS > OC and MS > MC were performed by computing average activations from first level parameter estimates and determining significance using *z* > 3.1, *p* = 0.05, (cluster-corrected) using mixed effects models to allow for population-wide inference. All voxels only associated with visual processing of complex stimuli were masked out by using the mask created from contrast VS > VC. This was done because differences between stereognosis and control conditions might have arisen from differences in visual stimulus complexity alone. All analyses were performed by using FMRIB's local analysis of mixed effects (FLAME).

In order to identify effector-independent areas of activation associated with spatial analysis and manipulation of objects in the hand and in the mouth, we performed a conjunction analysis $\left(OS>OC\right)\cap \left(MS>MC\right)$ on the respective group effects, using command-line scripts provided with FSL (easythresh_conj.sh), testing the “Conjunction Null Hypothesis” [23]. Here, only those voxels were identified that were significantly activated on a group level in each of both modalities (not *either/or*). All coordinates are reported in MNI space. Anatomical structures were identified using the Harvard–Oxford Cortical and Subcortical anatomical atlas while cytoarchitectonic structures and areas were identified using the Jülich Histological Atlas, all supplied with FSL software (https://fsl.fmrib.ox.ac.uk/fsl/fslwiki/Atlases). We calculated the probabilities of an activation being located in a particular cytoarchitectonic map using the probabilities provided by the respective histoarchitectonic map (Jülich Histological Atlas).

## Results

### Pre-Testing

In a previous behavioural experiment, we tested eight right-handed participants (5f, 3 m; mean age 31.0 years). It took approximately four seconds (mean 3.86 s; SD 1.96) to arrange a test piece in the default position.

### fMRI Experiment

#### Oral Stereognosis

The cerebral regions associated with oral stereognosis (contrast OS > OC) were found in predominantly left frontal and temporal areas, bilateral parietal areas, and the cerebellum (Table 1).

**Table 1:** Neural activations associated with oral stereognosis (mm)

Area Harvard-Oxford	%	Area Jülich	%	Side	*x*	*y*	*z*	*z*-value
Cerebellum		–		R	30	− 46	− 26	6.06
		–		R	6	− 64	− 38	6.04
		–		R	42	− 54	− 32	5.96
Caudate	51.8	Callosal body	41	R	10	18	− 2	3.94
MI	43	Broca’s area	36	L	− 48	6	32	3.73
	22	PMC	35	L	− 56	10	36	4.06
	22		64	L	− 58	2	42	3.86
SI	57	SI	76	L	− 50	− 28	46	4.68
	46		54	L	− 54	− 26	42	4.67
	25	SPL (aIPS)	20	R	34	− 34	40	4.44
IPL (aSMG)	42	IPL (PFt)	60	L	− 58	− 26	36	4.97
	77	IPL (PF)	58	R	62	− 24	42	4.61
	45	IPL (PFt)	50	R	50	− 30	44	4.43
pMTG	56	–		L	− 62	− 36	− 14	4.13
	79	–		L	− 68	− 34	− 10	3.98
pITG	38	–		L	− 58	− 40	− 16	4.04

Nomenclature of cytoarchitectonic maps of the inferior parietal lobule taken from Caspers et al. [24]

*Harvard-Oxford* Harvard-Oxford cortical and subcortical structural atlas, *Jülich* Jülich histological atlas, *R* right, *L* left, *MI* primary motor area, *PMC* primary motor cortex, *SI* primary somatosensory cortex, *SPL* superior parietal lobule, *aIPS* anterior intraparietal sulcus, *IPL* inferior parietal lobule, *aSMG* anterior supramarginal gyrus, *pMTG* posterior middle temporal gyrus, *pITG* posterior inferior temporal gyrus

Significant clusters of activation were characterized by their anatomical localization (Harvard-Oxford Cortical and Subcortical Structural Atlas/ Jülich Histological Atlas) and their MNI coordinates (x, y, z) in mm. % = probability of given cytoarchitectonic area

Significant bilateral activations (*z* > 3.1, *p* = 0.05) were found in the inferior parietal lobule (IPL)—particularly in the anterior supramarginal gyrus (aSMG) and the primary somatosensory cortex (SI).

Furthermore, we observed significant right hemispheric activations in the cerebellum, the caudate, the superior parietal lobule (SPL), as well as in the left premotor cortex (PMC). Further areas showing significant left activations were the primary motor area (MI) and the posterior parts of the middle temporal gyrus (pMTG) and the inferior temporal gyrus (pITG). Accordingly, statistical *z*-value maxima within clusters of significant activation (*z* > 3.1, *p* = 0.05, cluster-corrected for multiple comparison) were observed in the right and the left hemisphere. The two areas with the highest *z*-values were the right part of the cerebellum and the left IPL (Table 1).

In summary, neural activations were found predominantly in frontal (PMC, MI), parietal (SI, IPL) and subcortical areas (caudate, superior cerebellum). Additionally, there were activations in temporal areas (pMTG, pITG).

#### Manual Stereognosis

The cerebral regions associated with manual stereognosis (contrast MS > MC) were found in bilateral frontal, parietal and occipital areas, and the left thalamus.

Significant bilateral activations (*z* > 3.1; *p* = 0.05) were observed in the PMC, the posterior part of the superior frontal gyrus (pSFG), and the posterior part of the middle frontal gyrus (pMFG). Significant bilateral activations were also present in the SPL—including the left anterior part of the intraparietal sulcus (aIPS) and in the superior part of the lateral-occipital cortex (sLOC). Furthermore, there were significant activations in the left thalamus. Accordingly, statistical *z*-value maxima within clusters of significant activation (*z* > 3.1, *p* = 0.05, cluster-corrected for multiple comparison) were observed in the right and the left hemisphere. The two areas with the highest *z*-values were observed in the SI and the SPL (aIPS) in the left hemisphere (Table 2). No cerebellar activations were seen.

**Table 2:** Neural activations associated with manual stereognosis (mm)

Area Harvard-Oxford	%	Area Jülich	%	Side	*x*	*y*	*z*	*z*-value
Thalamus	90.5			L	− 18	− 30	0	5.2
pMFG	45	–		R	32	0	60	4.24
	32	PMC	27	L	− 26	− 2	54	5.01
	23		10	L	− 32	0	48	4.58
pSFG	37	PMC	31	L	− 26	− 4	62	4.88
	57	PMC	18	R	24	4	64	4.13
	24	PMC	20	R	28	− 4	56	5.45
SI	32	aIPS	22	L	− 38	− 36	42	6.74
SPL	37	SI	48	R	34	− 46	70	5.31
	49	SPL (7PC)	70	R	28	− 50	56	4.7
	54	SPL (7PC)	15	L	− 28	− 50	52	5.92
sLOC	38	SPL (7A)	55	R	26	− 58	68	5.15
	32	–		R	22	− 86	20	4.26
	19			L	− 24	− 76	20	5.53
	42		6	R	26	− 80	20	4.3

Nomenclature of cytoarchitectonic maps of the inferior parietal lobule taken from Caspers et al. [24]

*Harvard–Oxford* Harvard–Oxford cortical and subcortical structural atlas, *Jülich* Jülich histological atlas, *L* left, *R* right, *pMFG* posterior part of the middle frontal gyrus, *PMC* primary motor cortex, *pSFG* posterior part of the superior frontal gyrus, *SI* primary somatosensory cortex, *aIPS* anterior intraparietal sulcus, *SPL* superior parietal lobule, *sLOC* lateral-occipital cortex

Significant clusters of activation were characterized by their anatomical localization (Harvard–Oxford Cortical and Subcortical Structural Atlas/ Jülich Histological Atlas) and their MNI coordinates (*x*, *y*, *z*) in mm. % = probability of given cytoarchitectonic area

**Fig. 3:**
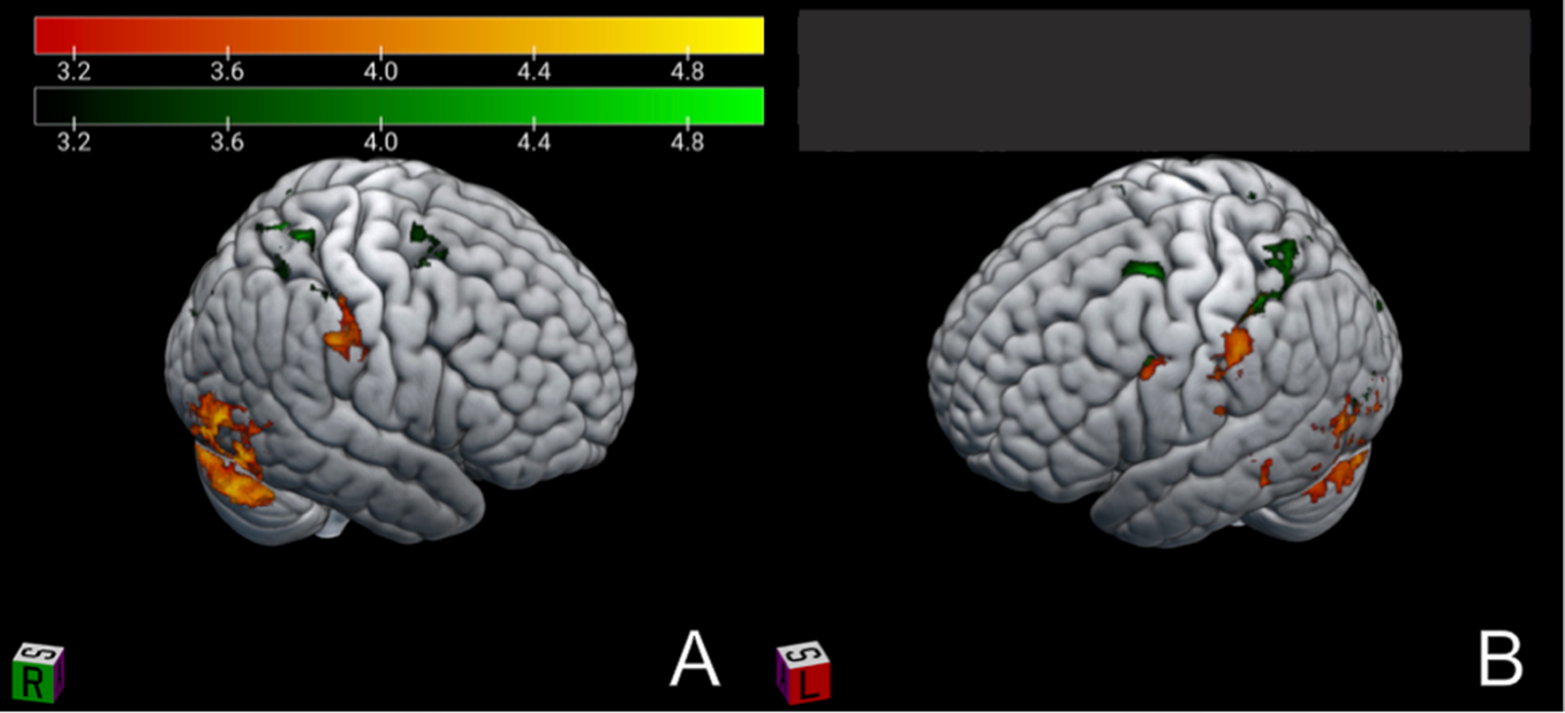
Foci of activation in oral (red) and manual (green) stereognosis of standard brain (MNI 152). *z* ≥ 3.1, *p* ≤ 0.05

Superimposition of both contrasts onto the MNI152 brain for visual purposes shows that distinct activations for each effector were found in two adjacent areas: the IPL for oral stereognosis and the SPL for manual stereognosis, reminiscent of the somatotopic organization of the human brain (Fig. 3).

#### Conjunction Analysis

Conjunction analysis revealed significant overlapping activations in parietal and temporo-occipital areas (file 2 of the Supplementary Material).

Significant overlapping neural activations were found in the SI, the IPL—including the aSMG and the aIPS-, the inferior part of the LOC (iLOC), the temporal occipital fusiform cortex and the temporo-occipital part of the inferior temporal gyrus (tITG). Neural activations were only present in the left hemisphere (Table 3).

**Table 3:** Conjunction of neural activations of oral and manual stereognosis (mm)

Area Harvard-Oxford	%	Area Jülich	%	Side	*x*	*y*	*z*	*z*-value
Temporal occipital fusiform cortex, iLOC	14			L	− 40	− 60	− 6	5.01
iLOC	61			L	− 50	− 66	− 2	4.75
	70		10	L	− 50	− 68	− 6	4.71
SI	35	SI	49	L	− 42	− 30	40	4.43
	41	IPL (PFt)	46	L	− 48	− 26	36	3.87
aSMG	56		52	L	− 54	− 30	42	3.59

Nomenclature of cytoarchitectonic maps of the inferior parietal lobule taken from Caspers et al. [24]

*L* left, *R* right, *vox* voxel, *Harvard-Oxford* Harvard-Oxford cortical and subcortical structural atlas, *Jülich* Jülich histological atlas, *iLOC* inferior lateral occipital cortex, *SI* primary somatosensory cortex, *IPL* inferior parietal lobule, *aSMG* anterior supramarginal gyrus

Significant clusters of activation were characterized by their anatomical localization (Harvard-Oxford Cortical and Subcortical Structural Atlas/ Jülich Histological Atlas) and their MNI coordinates (x, y, z) in mm. % = probability of given cytoarchitectonic area

In summary, overlapping neural activations were found in left temporo-occipital and left parietal areas.

## Discussion

In our study, we demonstrate for the first time that oral stereognosis, an ability that is crucial for successful swallowing, draws critically upon frontoparietal networks and potentially the cerebellum. The networks associated with manual tactile exploration and manipulation in our study are in good agreement with previously published literature [16, 18, 25, 26]. They thus underscore the general validity of our study.

Neural activations for oral spatio-tactile sensation and manipulation were found in frontal, parietal and temporal areas, and the cerebellum. In particular, activations were observed in the SI, the IPL (aSMG), the pMTG and pITG.

Crucially, the network areas associated with oral stereognosis are not exactly the same as those typically associated with manual stereognosis but are located caudally from them. This is in good congruence with the somatotopic organization of the sensorimotor cortices [27]. Still, both frontal and parietal foci of oral activation are in close relationship to the manual representations and share common areas of activation.

Activations in primary motor (premotor cortex (BA6L), primary sensory cortex bilaterally (BA2L + R), cerebellum and caudate nucleus are well explained by the increased demand placed on motor preparation, sensory feedback and fine-grained motor control in the stereognosis task. All these areas have been implied in oral and tongue non-object-related motor tasks previously [28–30]. In particular, higher activations in the cerebellum in an object-related task without strong spatial properties (vs. no object present) have been reported for an oral task in the literature [29]. Furthermore, the cerebellum has been described as relevant part of swallowing motor control in several studies non-invasive neurostimulation studies [31–33] These studies indicate an influence of the cerebellum on cortical swallowing modules [34]. Accordingly, we see strong activations in the oral stereognosis condition which places high demands on fine-grained motor control.

Still, we would argue that these areas (and cerebellar areas in particular) show higher activity due to higher complexity of the task without necessarily implying spatial cognition, because they do not show up in the conjunction analysis designed for highlighting areas involved in stereognosis. In order to properly test this hypothesis, a supporting experiment with a different control condition such as rapid diadochokinesis but not involving object manipulation would be intriguing.

The areas correlated with the particular aspect of stereognosis are highlighted by the conjunction analysis: Here, the left superior parietal cortex extending from primary somatosensory cortex into anterior intraparietal sulcus and left lateral occipital cortex, inferior division, is of particular interest. Left anterior intraparietal sulcus has been identified by Grefkes and colleagues in a series of experiments to subserve visuo-tactile transformations in a visuo-manual task [18, 35] following earlier work on macrogeometric features of manual perception [36]. This is in good congruence with primate studies that highlight the intraparietal sulcus as a crucial area for spatial calculations subserving object-related lip and finger movements in monkeys, e.g. as reviewed in [37].

Anterior parietal cortex (cytoarchitectonic areas BA2, PFt and hIP2) therefore could be the neuronal correlate for “feeling for action”, i.e. spatial analysis of an intraoral object for further manipulation, in analogy to “seeing for action” in the dorsal stream of sensory processing [38]. Similar functions have been described for this region in the manual domain [39] and for other effector-independent visuo-motor transformations [40].

Importantly, it has been shown that lateral occipital cortex, on the other hand, also holds supramodal (visual and tactile, but not auditory) representations of graspable objects [41, 42] in the ventral stream of sensory processing, even if tactile information only is present. In the context of the current study, this area could provide the substrate for a mental object representation with respect to visual shape more than action-relevant shape. It thus can be argued that both inferior parietal cortex and lateral occipital cortex hold effector-independent, spatially coded object representations that are necessary for object recognition (ventral stream) and manipulation (dorsal stream) by both mouth and hand.

As for lateralization, there seems to be a dominance for the left hemisphere in our data, particularly for the conjunction term. On visual inspection of the data, it becomes clear, however, that activations are bilateral for all conditions, but show a slight left-hemispheric dominance for all stereognosis conditions. A similar pattern has been shown for directional tongue movements without an object by Watanabe et al. [43]. Left-hemispheric predominance of the manual stereognosis condition must be explained, however, with the fact that this task was performed with the right hand. Accordingly, it has been described previously that lesions of the parietal cortex lead to tactile apraxia and loss of stereognosis in the contralesional hand [44, 45]. Therefore, we cannot make firm inferences on lateralization of oral stereognosis from our data.

A potential limitation could arise from the fact that slightly differently sized test pieces were used. This cannot explain our main findings of our study, however. Basic physical properties of an object are represented cortically in primary and secondary sensory cortical areas (SI and SII), mainly [16, 46]. Therefore, the activation observed in SI could potentially be elicited by a size difference. Crucially, however, activations in supramarginal gyrus and inferior parietal lobule in the oral condition are highly unlikely to be an artifact of differences in size of the test pieces, because here, we contrasted manipulation of equally sized objects in the oral conditions. Furthermore, tactile object memory has been shown to involve the parietal operculum, well apart from the activations observed by our study [47].

Still, our study improves on the only prior fMRI study [14] on oral stereognosis in several important ways: first of all, we introduced a control condition encompassing basic sensorimotor processing, irrespective of the spatial properties of the stimulus. This allowed us to better isolate stereognosis-specific neuronal activation by subtracting the basic motor condition from the stereognosis task. Secondly, Fuji and colleagues tasked their subjects with exploring complex stimuli and memorize the shape, so that subjects could later replicate the shapes by drawing the hitherto unknown stimulus on paper after scanning. This might explain why in this particular study, lots of activation in frontal and prefrontal areas can be seen, as these areas are usually associated with working memory and executive control. Finally, Fuji and colleagues did not perform comparative analyses, such as a conjunction between the two modalities. Yet, critical areas such as the transition zone between primary somatosensory cortex and secondary sensory areas were also shown to be active, in congruence with our present study.

## Implications

The current findings in healthy participants demonstrate both distinct and overlapping cortical areas of activation for oral and manual stereognosis in the human brain. While oral stereognosis shares many neuronal substrates with manual stereognosis, there seems to be a cranio-caudal gradient in neuronal representation. This finding could have immediate implications for our understanding of the complexities of the oral phase of swallowing, building on literature describing the neuronal correlates of primary sensory representation of the oral cavity [13]. In extension, it can serve as a physiological baseline from which to draw inference about failures of oral stereognosis as seen e.g. in stroke patients. It could also help explain why in Parkinson’s disease, where the parietal cortex usually is spared until late in the disease [48], no impairment of stereognosis could be found [9–12]. It might be of interest to examine stroke patients in more detail, e.g. include lesion studies, and to perform studies on the neuronal representation of oral stereognosis in healthy aging and neurodegenerative disorders with a predilection for the parietal cortex, such as Alzheimer’s disease (AD). Especially in AD dysfunction of the oral stage of swallowing such as prolonged bolus preparation and prolonged oral transit time is frequent [49]. Thus, examining the abilities of oral stereognosis in AD patients and correlating it with findings of instrumental swallowing diagnostics could create a better understanding of the underlying pathophysiology. However, the oral stage of swallowing is not only affected by perceptual and spatial deficits but also by loss of taste and olfactory function, which is common in patients with dementia [50]. Investigating the respective contribution of these additional sensory modalities could be interesting for future studies, as it is unique to swallowing in comparison to manual stereognosis where there is no taste information.

## Supplementary Information

Below is the link to the electronic supplementary material.

Supplementary file1 (TIFF 3699 kb)—Example for visual presentation in the experimental block

Supplementary file2 (TIFF 13658 kb)—Conjunction analysis. Coronal and transversal section of standard brain (MNI 152). *z* ≥ 3.1, *p* ≤ 0.05

## Data Availability

Due to local data management policies, data and code are available upon reasonable request by qualified researchers after meeting the following requirements: A formal data sharing agreement and formal project outline. Approval from the requesting researcher's local ethics committee. Agreeing to co-authorship for resulting publications.
