# Supplementary figures and images for: Neural Correlates of Oral Stereognosis—An fMRI Study
Source: Dysphagia. 2022 Sep 10;38(3):923–32. doi: 10.1007/s00455-022-10517-2 (PMC10182931; doi:10.1007/s00455-022-10517-2)

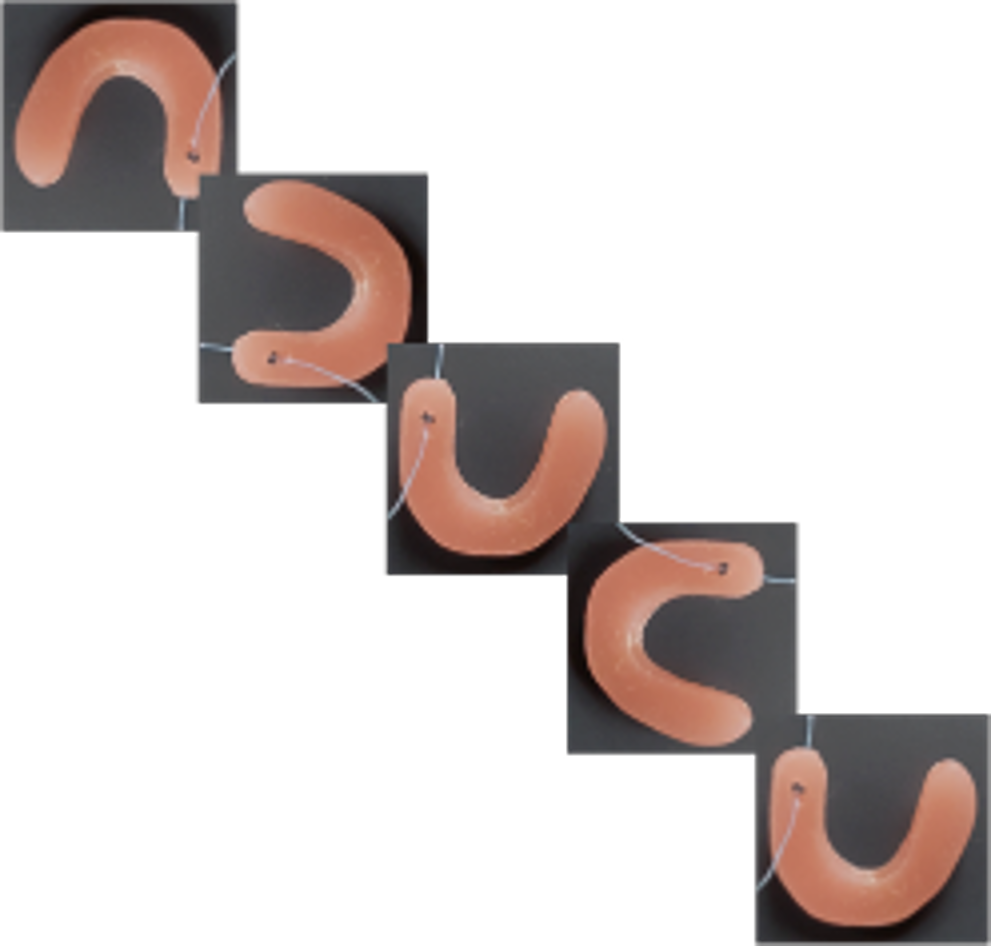

Supplement: Supplementary file 1 — Supplementary file1 (TIFF 3699 kb)—Example for visual presentation in the experimental block [file 455_2022_10517_MOESM1_ESM.tiff]

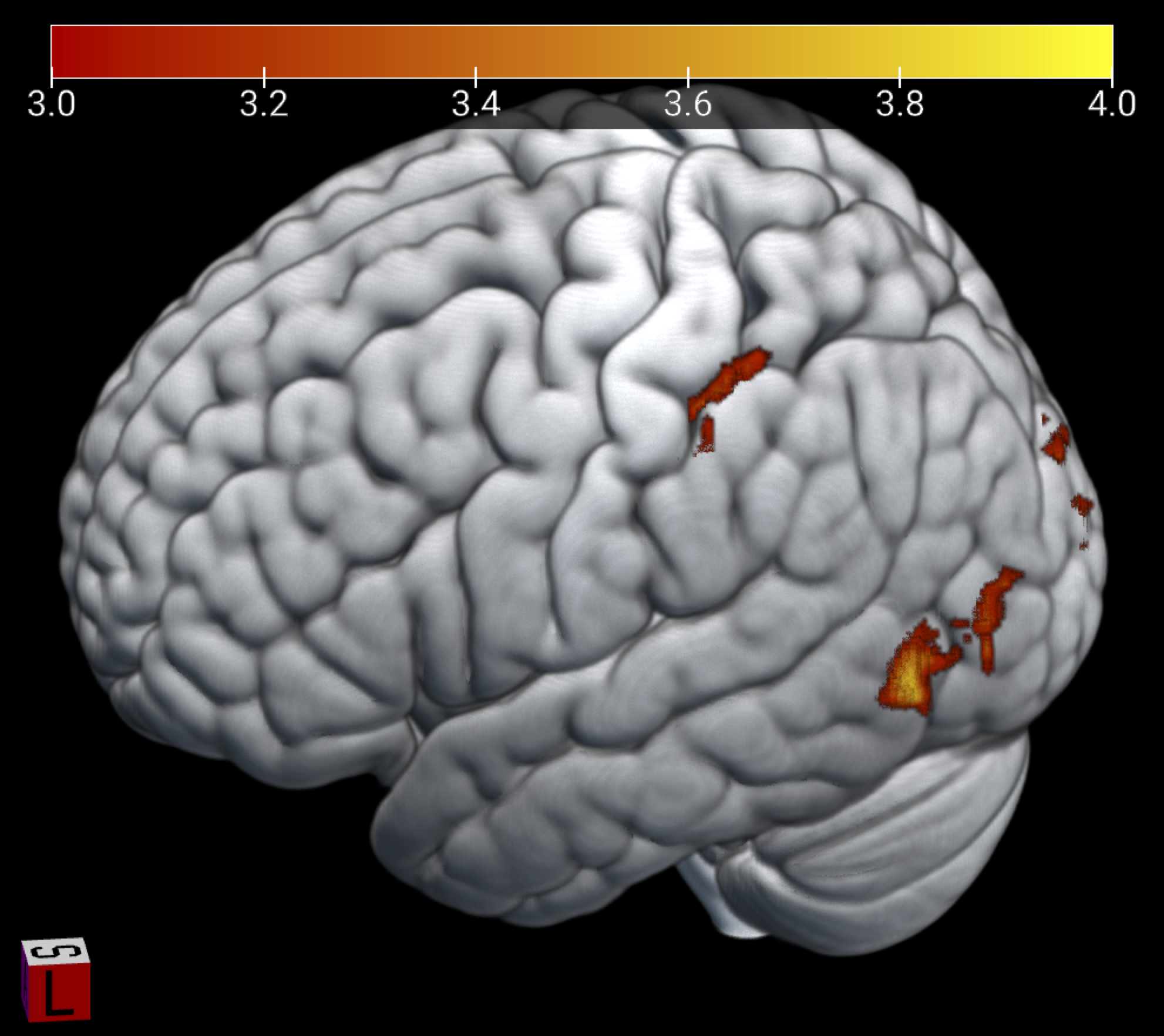

Supplement: Supplementary file 2 — Supplementary file2 (TIFF 13658 kb)—Conjunction analysis. Coronal and transversal section of standard brain (MNI 152). z ≥ 3.1, p ≤ 0.05 [file 455_2022_10517_MOESM2_ESM.tiff]
